# Photoacoustic Imaging with Capacitive Micromachined Ultrasound Transducers: Principles and Developments

**DOI:** 10.3390/s19163617

**Published:** 2019-08-20

**Authors:** Jasmine Chan, Zhou Zheng, Kevan Bell, Martin Le, Parsin Haji Reza, John T.W. Yeow

**Affiliations:** 1 Department of Systems Design Engineering, University of Waterloo, Waterloo, ON N2L 3G1, Canada; 2 Department of Physics, University of Waterloo, Waterloo, ON N2L 3G1, Canada

**Keywords:** photoacoustic tomography, capacitive micromachined ultrasound transducer, photoacoustic endoscopy, photoacoustic microscopy, photoacoustic computed tomography

## Abstract

Photoacoustic imaging (PAI) is an emerging imaging technique that bridges the gap between pure optical and acoustic techniques to provide images with optical contrast at the acoustic penetration depth. The two key components that have allowed PAI to attain high-resolution images at deeper penetration depths are the photoacoustic signal generator, which is typically implemented as a pulsed laser and the detector to receive the generated acoustic signals. Many types of acoustic sensors have been explored as a detector for the PAI including Fabry–Perot interferometers (FPIs), micro ring resonators (MRRs), piezoelectric transducers, and capacitive micromachined ultrasound transducers (CMUTs). The fabrication technique of CMUTs has given it an edge over the other detectors. First, CMUTs can be easily fabricated into given shapes and sizes to fit the design specifications. Moreover, they can be made into an array to increase the imaging speed and reduce motion artifacts. With a fabrication technique that is similar to complementary metal-oxide-semiconductor (CMOS), CMUTs can be integrated with electronics to reduce the parasitic capacitance and improve the signal to noise ratio. The numerous benefits of CMUTs have enticed researchers to develop it for various PAI purposes such as photoacoustic computed tomography (PACT) and photoacoustic endoscopy applications. For PACT applications, the main areas of research are in designing two-dimensional array, transparent, and multi-frequency CMUTs. Moving from the table top approach to endoscopes, some of the different configurations that are being investigated are phased and ring arrays. In this paper, an overview of the development of CMUTs for PAI is presented.

## 1. Introduction

Photoacoustic imaging (PAI) is a developing imaging technique researched for various clinical applications, including oncology [1], neurology [2,3,4], dermatology [5,6], and ophthalmology [7]. In PAI for biomedical application, a short pulse laser, usually in the visible to near-infrared of the electromagnetic spectrum is first illuminated onto the tissue. The tissue then experiences thermoelastic expansion where it heats up and expands. During this brief expansion, the pressure within the tissue increases, thereby generating ultrasound waves that are picked up by the detector. The detector converts this ultrasound wave into a signal which is amplified and stored into a data acquisition card for image reconstruction [8].

The strength of PAI lies in its ability to bridge the gap between pure optical and acoustic imaging henceforth producing images with the resolution of submicrometer at penetration depth as deep as several centimeters. The working principle of optical imaging is mainly governed by the scattering and absorption of photons, which can be categorized into four regimes. The ballistic regime is the region within the mean free path where the photons have not gone through any significant scattering. One example of an imaging system in this regime is confocal microscopy. In the quasi-ballistic regime, the region between the mean free path to the transport mean free path, the photons sustain minimal scattering. Nevertheless, this has a negligible impact on the photon’s memory of the original path. Just below the quasi-ballistic regime is the quasi-diffusive regime. In this regime, the photons are subjected to much scattering such that the spatial and temporal coherence is degraded. Beyond 10 times the transport mean free path is the diffusive regime. The photons in this regime have a negligible recollection of the original path [8]. The spatial and temporal coherence is completely lost in this regime. For biological tissue, the mean free path is on the order of 0.1 mm while the optical transport mean free path is the order of 1 mm [8].

On the other hand, acoustic imaging makes use of the acoustic wave to penetrate deeper than the optical imaging. Imaging is done based on acoustic contrast mismatch, which, in the case of biomedical imaging is related to the mechanical properties of soft tissues [9]. As acoustic impedance is not encoded with molecular information, therefore acoustic imaging is unsuited for monitoring of molecular activities such as the oxygenation level in hemoglobin [10]. Moreover, there is a trade-off between the penetration depth and the resolution where a high-resolution image commonly yields a low penetration depth and vice versa. This trade-off exists because the resolution produced by the acoustic imaging is dependent on the frequency of the signal. A shorter pulse length is usually desirable as it gives a higher image resolution. However, with high frequency comes high ultrasonic attenuation [9,11]. Consequently, it restricts the acoustic wave from penetrating deeper into the tissue.

Taking advantage of the optical contrast and improved acoustic penetration PAI can penetrate deeper and produce higher resolution images in comparison to what is achievable by pure optical and acoustic imaging techniques. Unlike optical imaging, PAI does not need to take into account the scattering of the returning light and instead relies on the acoustic wave to form the image. This allows the PAI to produce high-resolution images that cover beyond the quasi-ballistic regime. By detecting acoustic waves instead of photons, PAI has a depth to resolution ratio of 2 orders of magnitude higher than the common optical imaging methods [12,13,14]. Furthermore, PAI is very sensitive to small changes in the optical absorption variation and can fully reflect these changes on the amplitude of the photoacoustic signal [15]. This linear relationship between the signal and the optical absorption permits linear spectral unmixing for multiplex and functional imaging [10].

Employing the idea that every biological cell and tissue have different optical absorption coefficients, an appropriate wavelength can be selected for the specific target. Hemoglobin is one of the strongest optical absorbers in the visible light range [10,16]. When a tumor develops in the body, the amount of blood flow in that region tends to be higher as more blood vessels grow to provide cancer cells with oxygen and nutrients. This is known as angiogenesis. With PAI, a specific wavelength, such as 532 nm, can be used to track hemoglobin. Thus, areas with high blood flow can be detected without exogenous label [16]. The ability to image hemoglobin will be immensely useful in aiding clinicians in the advancement of cancer studies. Other examples of endogenous contrast include melanin, lipids, and DNA/RNA [17]. Exogenous contrast can be introduced to target specific cell or tissue when endogenous contrast is unable to produce a substantial signal. With a large variety of exogenous contrast available, selecting an exogenous contrast with a high absorption coefficient at the specified laser wavelength can improve the imaging sensitivity. Examples of exogenous contrast used in PAI are organic dyes, fluorescent proteins, and nanoparticles [17].

Two major components for PAI are the photoacoustic signal generator and the detector. The generator is usually the pulse laser which is used to irradiate the sample. Pulse lasers are normally preferred as pulse excitations to produce a better signal to noise ratio (SNR) than continuous-wave excitations [14]. Some excitation capabilities of the pulse laser that dictate the spatial characteristic and contrast include the pulse width, pulse length, and wavelength of the laser [18,19]. The detector, on the other hand, assumes the role of receiving the acoustic signal that was generated by the illuminated sample. The development of novel detection mechanisms provides additional routes toward improving image quality by offering higher sensitivity, resolution, and SNR. The four key parameters of the detector, which can affect the image quality are the center frequency, bandwidth, sensitivity, and size [20]. In photoacoustic imaging, the detector’s center frequency and bandwidth are two of the most important parameters that determine the image resolution. In order to attain images with micrometers resolution, a detector needs to have a center frequency and bandwidth that is in the MHz range. As mentioned previously, a higher image resolution will normally entail a smaller penetration depth and vice versa. Thus, the appropriate center frequency and bandwidth need to be selected based on the intended application. Another factor to consider when it comes to choosing a detector is sensitivity. A common measure to determine sensitivity is the noise-equivalent-pressure (NEP). NEP is generally defined as the amplitude of the detected pressure with respect to the noise level. A detector that is able to pick up a signal in the Pa or sub-Pa range is usually needed to detect photoacoustic signals resulting from low excitation laser fluences or deeper penetration depths in turbid media such as biological tissues [20]. In designing a PAI system, especially for applications such as minimally invasive surgery (MIS) endoscopes, size is a critical component. Catheters, frequently used for MIS applications, highlight size constraints. Catheters are long tubes with diameters of about 0.5–3 mm hence, a rule of thumb for detectors used for MIS application is that they should have the ability to operate at these device scales [21].

The more commonly used detectors in PAI are Fabry–Perot interferometers (FPIs), micro ring resonators (MRRs), piezoelectric transducers, and capacitive micromachined ultrasound transducers (CMUTs). Some issues with using only a single sensor element for imaging are the reduction in imaging speed and the inevitable formation of motion artifacts. One solution to these issues is to fabricate the sensor elements into an array [22]. However, this can be challenging for the FPI as calibration is required at every interrogation location. Fabricating MRR into an array is also difficult because of optical coupling. The fabrication techniques used for piezoelectric transducers and CMUTs have made fabricating them into an array less of an issue. Since mechanical dicing is used in the fabrication of most traditional piezoelectric transducer arrays, there is a limit on how small the pitch can be. This then affects the frequency of the transducer which in turn determines the resolution of the image. The restriction in the pitch dimension consequently produces a lower image resolution. CMUTs, on the other hand, do not need mechanical dicing in its fabrication process. Therefore, higher frequency CMUT arrays can be made to produce higher image resolution. On top of that, CMUT’s fabrication technique has allowed it to be more customizable to meet design specification [23].

The first reported CMUTs were presented by a research group at Stanford in 1994 with the goal of making an airborne ultrasound transducer that can operate in the MHz range [24,25]. The motivation to further develop CMUT for other application resulted from an immersion test where it has shown that CMUTs can provide a larger bandwidth over traditional piezoelectric transducers [25]. Since then CMUTs have been used in a variety of domains ranging from high-intensity focused ultrasound [26,27], ultrasound imaging [28,29,30,31,32,33], sensing application [34,35] to non-destructive testing [36]. Some of these applications and CMUT designs have been further adapted to advance CMUTs for PAI applications.

This review paper covers the concepts and development of CMUTs for PAI. In Section 2, the different PAI modalities are introduced while in Section 3, some of the detectors that are currently used for PAI are presented. Lastly, Section 4 discusses the current developments of using CMUTs for PAI.

## 2. Photoacoustic Modalities

PAI can be split into two main categories based on the way images are formed. Photoacoustic computed tomography (PACT) uses reconstruction-based image formation, while photoacoustic microscopy (PAM) uses focused-based image formation.

### 2.1. Photoacoustic Computed Tomography

In PACT, an unfocused optical beam is used to excite an area of the material, and an array of sensors measure the generated ultrasound waves in the various positions [15]. PACT has reportedly been able to penetrate as deep as 7 cm into the tissue, but consequently, it may only deliver a lateral resolution of hundreds of micrometers [37,38]. With different algorithms available, time reversal and back-projection methods are the two most popular techniques for image reconstruction. Time reversal is a time iterative reconstruction algorithm and as its name suggests, works by re-emitting the wave in a time-reversed manner via an acoustic propagation model [10,39]. Because of the intensive computation required to obtain the image, the time-reversal method has not been applied for practical use [10,13,40]. The back-projection method, like time reversal, employs the knowledge of the speed of sound in the medium to resolve the ultrasound waves and back project them to form the image [10]. This method is similar to the delay and sum beamforming method used in ultrasound imaging. Since the back-projection method requires lesser time to form an image, it is more applicable for real-time imaging [41].

PACT can be used for many applications ranging from microscopic to macroscopic imaging [37]. As it receives the signal from multiple elements, PACT can provide a larger field of view (FOV) with a single shot in the diffusive regime. Therefore, it can be used to image the whole body of small animals [38] and has also been evaluated for uses in neurology [2,4,42,43], cardiology [44], and label-free breast cancer studies [45]. However, imaging such a large field of view requires a large acceptance angle to receive signals from multiple locations of the same object [15]. With such a large dataset, the imaging speed will be dependent on the data acquisition system [12].

### 2.2. Photoacoustic Microscopy

Unlike PACT, PAM uses a focused beam and detects using a single element that is raster-scanned about the sample [46]. The signal which is dependent on the optical energy deposited is resolved with respect to the acoustic axis, and the envelope of this recorded time-domain signal is extracted to form the image [15]. PAM is generally used for applications that require high-resolution images rather than deep penetration depth like the studying of the microenvironment of diseases using small animals [47]. PAM can be further divided into two imaging methods depending on whether the generated photoacoustic signal is more optically or acoustically focused. Optical resolution photoacoustic microscopy (OR-PAM) takes advantage of the optical focus to obtain a high lateral resolution, while acoustic resolution photoacoustic microscopy (AR-PAM) utilizes acoustic focusing to image at depths greater than the transport mean free path of the excitation pulse.

#### 2.2.1. Acoustic Resolution Photoacoustic Microscopy

As mentioned previously, AR-PAM has a tighter acoustic focus for imaging. Since the acoustic focus is limited by the acoustic diffraction limit rather than the optical diffraction limit, the resolution attainable by AR-PAM is only tens of micrometers [48]. Conversely, acoustic waves scatter less than visible photons in scattering media. Hence, AR-PAM has been demonstrated to penetration depths of up to 11 mm [49], which is 10 times more than OR-PAM.

The maximum permissible exposure (MPE) allowed by the American National Standard Institute (ANSI) for nanosecond pulsed wavelengths between 400 to 700 nm in human tissue is 20 $\mathrm{mJ}/{\mathrm{cm}}^{2}$ [50]. As a larger area is illuminated, AR-PAM has a higher energy allowable limit than OR-PAM [12]. This allows more photons to reach deeper into the tissue, producing higher resolution images at deeper penetration depths. Moreover, AR-PAM is able to maintain the same contrast for a broad range of imaging resolution [51]. These advantages make AR-PAM a good imaging modality to be explored for different applications such as imaging sentinel lymph nodes [49,52] detecting cystography [53], and gastrointestinal imaging [54].

#### 2.2.2. Optical Resolution Photoacoustic Microscopy

OR-PAM, on the other hand, emphases more on the photons rather than the acoustic waves. In OR-PAM, the optical focus of the excitation beam is tightened to form the image. The resolution is limited by the optical diffraction of the focused laser onto the tissue [55,56]. Indeed, OR-PAM is able to produce images with higher resolution than AR-PAM, but its penetration depth is restricted by the optical transport mean free path, which for biological tissue is ~1 mm [55].

Making use of its optical focus, OR-PAM can be used for label-free imaging within the optical quasi-ballistic regime. The high resolution that it can achieve makes it suitable for imaging small targets ranging from a capillary vein, which can be less than 10 μm to even smaller targets like cells [15,46,48]. OR-PAM has also been investigated for a broad range of applications to understand diseases and the biological environments in the eyes [57,58], skin [59], and brain [60].

### 2.3. Detectors Used in the Different PAI Modalities

There are three detection geometries used in PACT applications namely, planar, cylindrical, and spherical. Planar view PACT systems usually use two-dimensional (2D) planar, phased, or linear arrays for imaging, while cylindrical view PACT systems detect with ring arrays [61]. Spherical view PACT systems have been used for imaging with arc-shaped transducer array [62] and hemispherical transducer array [44]. The axial resolution of all three PACT systems is determined by the bandwidth of the detector, while their lateral resolution is dependent on their detection geometries [14]. Similarly, the axial resolution of PAM is also determined by the bandwidth of the detector however, the lateral resolution is based on the acoustic wavelength for AR-PAM and optical wavelength for OR-PAM [17]. OR-PAM mainly relies on the optical focus to attain a high-resolution image while in AR-PAM, the laser beam is weakly focused and a focused transducer is used [63]. Nonetheless, higher frequency detectors are needed in both PAM modalities as their applications usually require higher resolution at smaller penetration depth. The resolution and penetration depth that various PAI modalities can deliver, along with their main area of applications are presented in Table 1.

## 3. Detectors

In this section, the four most common types of acoustic sensors used in PAI are discussed namely, FPIs, MRRs, piezoelectric transducers, and CMUTs. Leveraging on their inherent property of larger bandwidth, FPIs and MRRs can produce images with higher resolution than piezoelectric transducers and CMUTs. Nevertheless, piezoelectric transducers and CMUTs can be made into an array which helps to reduce imaging time and minimize the formation of motion artifacts. The ability to be fabricated into an array makes them suitable for high-resolution and real-time imaging applications. In addition to that, CMUTs can be integrated with electronics, which reduces losses because of parasitic capacitance. Further details and explanations of these different sensors are discussed in the following subsection.

### 3.1. Fabry-Perot Interferometer

**Figure 1:**
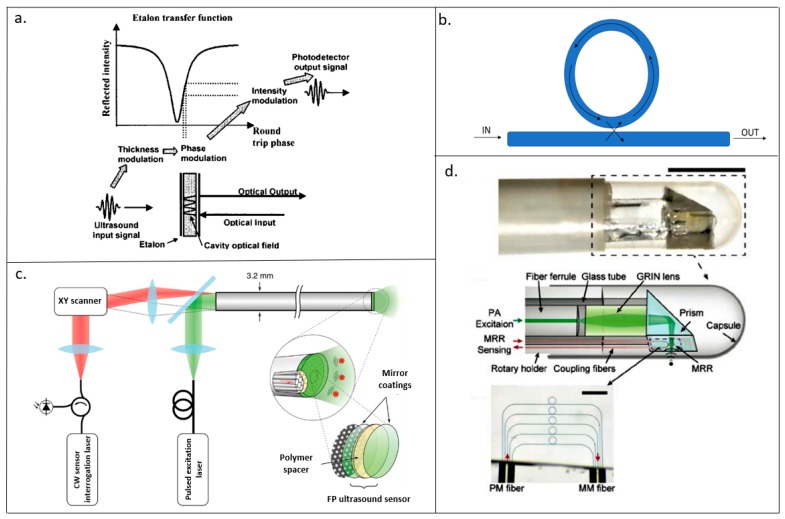
(**a**) Diagram of the working principle of Fabry–Perot interferometers (FPI). An incoming ultrasound wave causes a variation in thickness which in turn results in a phase modulation (Reproduced from [88], with the permission of AIP Publishing.); (**b**) schematic diagram of an micro ring resonators (MRR); (**c**) schematic diagram of a forward-viewing photoacoustic probe for endoscopy imaging used in [69]; (**d**) photoacoustic endoscopy with a MRR detector used in (Adapted with permission from ref [82], [The Optical Society]).

FPI is a type of interferometer that is made up of a thin transparent film sandwiched between two reflectors [65]. The pressure changes created by the incoming ultrasonic wave causes variation in the thickness and elasto-optic of the structure, which in turn, causes an optical phase shift [65]. An interferometer then transforms this optical phase shift into measurable intensity modulation [66]. The working principle of FPI is illustrated in Figure 1a.

FPI has been used as a detector for countless PAI applications [67,68,69,70]. Ansari et al. [69] designed a forward-looking photoacoustic endoscopy with an FPI array as shown in Figure 1c. Fabry–Perot films were deposited on the distal end of each of the 50,000 12-μm fiber core in an image guide bundle which were then placed into a 3.2-mm diameter 76-mm long probe. The acoustic sensor was found to have a 3-dB bandwidth of 34 MHz. 3D imaging of the different phantoms such as synthetic hair, leaf, duck embryo, and mouse skin were demonstrated. In the duck embryo, ex vivo imaging of microvasculature was done at a depth of 1 mm and vessels with diameters as small as 50 μm were observed. Vessels with diameters ranging from 53–180 µm were captured 2 mm deep inside the mouse skin.

FPI has moved on from laboratory experiments to clinical trials. Plumb et al. [70] built a PAI system to identify peripheral arterial disease using small and large vessels. Large vessels can be detected with current magnetic resonance imaging and ultrasound technology; however, these systems cannot be used to image small vessel which is essential for the diagnosis of diseases such as diabetes. In this study, the system imaged reflex vasoconstriction in the fingertips and the dorsalis pedis, a peripheral leg artery. A total of 8 volunteers underwent the dorsalis pedis imaging while 13 volunteers had their index finger imaged. The spatial resolution obtained with this system was 75–125 μm with a 3-dB bandwidth of 30 MHz.

As FPI sensitivity is not significantly affected by the element size, it can be made using smaller elements so much so that it can be approximated as a point detector, producing sharper images [71]. This can be seen in the previously reported systems [69,70] where the images produced showed a lateral resolution of tens of micrometers. Since Fabry–Perot sensors can be optically transparent to the excitation beam, they can be used for both transmission and reflection mode imaging applications [72]. Other advantages of FPI that contribute to the high-resolution images include being more sensitive and less susceptible to electrical noises [73]. That said, one major downside of FPI is the need to do mechanical raster scanning as typically only one FPI element is used for imaging. This means that longer imaging time is required for data collection [71]. In the above photoacoustic endoscopy experiment by Ansari et al. [69], it took about 25 min to form the image. The longer imaging time is due to the need for optical wavelength tuning at every interrogated location. These devices have been used for diverse purposes such as a temperature sensing [74,75,76], pressure sensing [76,77], sensing the concentration of biochemicals [78], and imaging of blood vessels [79].

### 3.2. Micro Ring Resonator

MRR comprises of a straight waveguide coupled closely together with a ring waveguide, shown in Figure 1b. The straight waveguide acts as the input and output port of the detector, while the ring waveguide acts as the resonator [80]. Like FPI, the incoming acoustic wave causes a change in the dimension and elasto-optic modulation of the ring waveguide, which in turn alters the refractive index [81]. The deviation in the optical path length because of the changing refractive index also induces a shift in the resonant frequency [82]. This shift in resonant frequency is detected by the photodiode, which gives a voltage output that is proportional to this shift in the resonant frequency of the acoustic wave.

Dong et al. [82] came up with an MRR-based photoacoustic endoscopy that was able to perform volumetric imaging on two phantoms; a 3D printed hollow black plastic tube and strands of hair taped and rolled together into a small tube. The two ends of the MRR were carefully attached to the fibers to form the photoacoustic probe as shown in Figure 1d. The probe was mounted on a homemade shaft for circumferential scanning and a linear motorized stage was used for axial scanning. The photoacoustic endoscope had a noise-equivalent pressure (NEP) of 35.2 Pa. The axial, tangential, and radial resolution obtained was 16.0 μm, 15.7 μm, and 4.5 μm, respectively. One major consideration for this endoscope was that the imaging performance was dependent on the stability of the MRR’s sensitivity during scanning. Another limitation of this endoscope design was circular scans as that would change the dimension of the fiber and cause an optical phase shift.

Unlike FPI, the thickness of the film affects the detector’s bandwidth instead of its sensitivity [55]. The sensitivity is, nonetheless, dependent on the size of the element. The small element size used in MRR makes wide angular response possible [55]. In fact, in some reports, MRR is found to have better sensitivity than FPI [55]. Since the element size is also related to bending loss, there is a restriction in the reduction of element size as this can impair the FOV [66]. Some other areas that MRR has been utilized are temperature sensing [83], biomedical imaging [84,85], and data transmission [86,87].

### 3.3. Piezoelectric Transducer

The piezoelectric effect is the conversion of kinetic energy due to mechanical stress to electrical energy, and vice versa. For years, piezoelectric transducers have been used for numerous purposes going from “sonar,” used in World War 2 to detect submarines [89] to modern-day applications such as non-destructive testing [90], energy harvester [91,92], and biomedical imaging [93,94,95,96]. Even though piezoelectric and photoacoustic effects were discovered in the 1800s, it was only in 1994 when the research of Kruger [97] reignited researchers’ interest in using PAI for biomedical applications. Since then, various researches were done in this domain. Throughout the years, more research has been done on the piezoelectric transducer to improve its performance. One area that researchers are investigating is the type of piezoelectric materials used for transducer. Various piezoelectric materials are being developed for transducers used in PAI applications including lead zirconate titanate (PZT) [98,99], polyvinylidene fluoride (PVDF) [100] and lead magnesium niobate-lead titanate (PMN-PT) [101]. Both PZT and PVDF based piezoelectric transducers have been commonly used for PAI applications [55]. PVDF has the advantage of lower acoustic impedance, while PZT offers a higher piezoelectric coupling coefficient. In this paper, the piezoelectric transducers discussed is based on the conventional PZT piezoelectric transducers.

Above 1 MHz, piezoelectric transducers have a higher sensitivity and lower thermal noise as compared to the other detectors [9]. Nonetheless, piezoelectric transducers have a limited frequency range. To make a high-frequency array, the transducer elements need to be placed close together to avoid aliasing. The pitch required for a 25 MHz transducer to avoid aliasing is approximately 30 μm [102]. As piezoelectric transducers are typically fabricated using mechanical dicing, it is difficult to keep the elements tightly packed to pitches less than 40 μm [21,102]. Nevertheless, some groups have managed to alter the conventional manufacturing process and fabricated high-frequency piezoelectric transducer arrays [103,104]. That said, the inherent properties of the piezoelectric transducer like the small bandwidth is still a constraint in producing high-resolution images. The need for an acoustic matching layer makes piezoelectric transducer bulkier [95]. Besides, piezoelectric transducers also face a huge problem in PAI as it is opaque, which can make delivering of light to the tissue a challenging task [71].

### 3.4. Capacitive Micromachined Ultrasound Transducer

**Figure 2:**
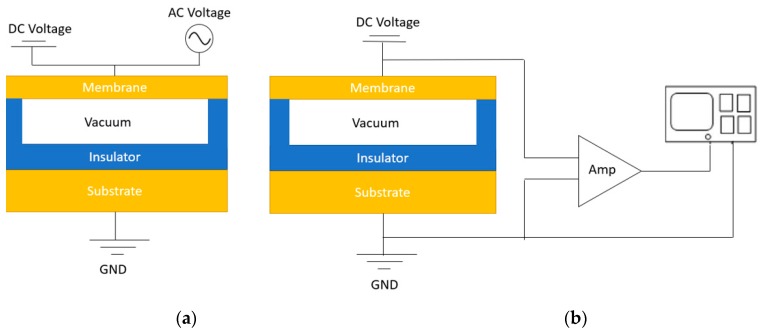
(**a**) Capacitive micromachined ultrasound transducers (CMUT) transmission mode; (**b**) CMUT receiving mode.

CMUTs, as its name suggests, are micro-electro-mechanical devices fabricated with micromachining, that can be used to receive and transmit ultrasound signals [32]. A typical CMUT cell consists of a membrane suspended over a vacuum gap. A thin layer of metal on top of the membrane forms the top electrode while the silicon substrate forms the bottom electrode [105]. An insulator is stacked on the silicon substrate in order to prevent the top and bottom electrodes from touching. In transmission mode, DC and AC voltages are applied to the electrodes. The DC voltage brings the top and bottom electrodes closer while the AC voltage actuates the membrane to produce an ultrasound signal. During receiving mode, only DC voltage is applied. The incoming sound wave modulates the gap height based on the wave frequency [106]. As a result, the capacitance of the membrane changes, thereby producing an output current. This output current is converted to a voltage signal and intensified by a transimpedance amplifier. Figure 2a shows the set-up of a CMUT in transmission mode while Figure 2b shows the CMUT in receiving mode. For PAI applications, CMUTs are mainly used as receivers. Therefore, they can be designed with the sole consideration of optimizing the receiving parameters.

Some design considerations in the fabrication of CMUTs include the diameter, the type of material, and the gap height, which can affect the performance of CMUTs. CMUTs can be modeled as a second-order system to obtain important parameters such as the resonant frequency and pull-in voltage. The resonant frequency is a critical parameter that determines the resolution of the image.

(1) $$w0=\;\frac{\frac{2.95t}{a^{2}}\sqrt{\frac{E}{\rho \;\left(1-\;v^{2}\right)}}}{\sqrt{1+0.67\;\frac{\rho_{m}a}{\rho t}}}$$

where *t* is the thickness of the membrane, *a* is the radius of the membrane, *E* is the Young’s modulus of the membrane material, v is the Poisson’s ratio of the membrane, $\rho_{m}$ is the density of the medium, and ρ is the density of the membrane. Another crucial parameter is the pull-in voltage. Pull-in voltage is the point where the electrostatic and the mechanical forces are equal, resulting in the top electrode snapping down on the substrate. Hence, it is important to operate CMUTs below the pull-in voltage. The pull-in voltage, $V_{pull\;in}$ can be found using

(2) $$V_{pull\;in}=\sqrt{\frac{8kg_{eff}{}^{3}}{27\varepsilon_{0}\varepsilon_{r}A}}$$

where $g_{eff}$ is the effective gap height and is calculated using $g_{eff}=\frac{t_{m}+t_{i}}{\varepsilon_{r}}+g_{0}$. $g_{0}$ is the original gap height, $t_{m}$ is the thickness of the membrane, $t_{i}$ is the thickness of the insulator, $\varepsilon_{r}$ is the relative permittivity of the insulator and the membrane material, $\varepsilon_{0}$ is the permittivity of free space, *k* is the spring constant, and *A* is the electrode area. Apart from the pull-in voltage, the gap height can also affect the sensitivity of CMUTs. More details and explanations can be found in [25].

### 3.5. Discussion of the Acoustic Sensors

Since FPIs and MRRs working principle is based on optical detection, they tend to have a higher image resolution as compared to piezoelectric transducers and CMUTs which operate based on acoustic detection. On the downside, the penetration depth of FPI and MRR is limited to millimeters. Table 2 shows the resolution and penetration depth attainable with the different detectors. Moreover, because of their delicate fabrication, it is difficult to fabricate FPI and MRR arrays. Some issues with using only a single sensor element for imaging are the reduction in imaging speed as observed in the above experiments and the inevitable formation of motion artifacts. One solution to these issues is to fabricate these sensor elements into an array [22]. The fabrication of FPI array is not as straight forward as fabricating piezoelectric transducer and CMUT arrays, additional steps are needed. After fabricating the FPI elements, these individual elements are placed onto a substrate or fiber one at a time to form the array. Careful calibration is required before the FPI array can be used for real-time imaging. For MRR, fabricating it into an array can be a challenging task as they rely on the optical coupling. On the contrary, a piezoelectric transducer can be made into an array and uses electronic scanning to produce the image [71,102]. Hence, piezoelectric transducer array does not face the issues that FPI and MRR encounter with mechanical raster scanning which makes it a more suitable candidate for real-time imaging.

The benefits of having piezoelectric transducer-based detectors over FPIs and MRRs apply to a CMUTs as well. Additionally, CMUTs have other advantages over piezoelectric transducers. With an acoustic impedance that is similar to tissues, CMUTs do not need a matching layer, making it less bulky. In general, the fractional bandwidth achievable by CMUTs are over 100% while piezoelectric transducers can typically only attain a fractional bandwidth of 60–80% [107]. As previously stated, one of the important parameters for PAI is the receiving sensitivity of the transducer [20]. Zheng et al. [107] compared the receive sensitivity of a 2.63 MHz CMUT and a 2.43 MHz piezoelectric transduce (Olympus, Waltham, MA, USA). The result showed that the CMUT element had a higher received sensitive of 22.57 mV/kPa while the piezoelectric transducer only attained a receive sensitivity of 4.28 mV/kPa. Furthermore, unlike piezoelectric transducer arrays, CMUT arrays are fabricated with photolithography instead of mechanical dicing. This has allowed the element pitch to be made smaller which in turn, contributes to a highly dense CMUT array that can produce high-frequency signals with minimal aliasing. It is difficult to accomplish these traits with the piezoelectric transducer. With micromachining, CMUTs can be easily made into any geometry and dimension to meet design specifications [23]. On top of that, micromachining is similar to the fabrication technique used for complementary metal-oxide-semiconductor (CMOS). Therefore, CMUTs can be easily integrated with electronics, integrated circuit (IC) which can help to reduce parasitic capacitance in electronics and improve the SNR [102]. The improvement in SNR can be seen from the experimental results attained by Vallet et al. [98]. The group did an experiment to compare the use of the PZT piezoelectric transducer and CMUT probes for PAI. In the experiment, 2.6 MHz (Vermon SA, Tours, France) and 3.2 MHz (ACULAB, Rome, Italy) CMUT probes, and 7.2 MHz (Prosonic Co., Gyeongsangbuk-do, Korea) and 7.3 MHz (Esaote S.p.A., Florence, Italy) piezoelectric transducer probes were used to image agar phantoms. Based on the results attained, the CMUT probes had a larger fractional bandwidth with an SNR of up to 14 dB and contrast to noise ratio (CNR) of up to 20 dB higher than piezoelectric transducers.

In recent years, piezoelectric material has also been used to fabricate micromachined ultrasound transducers. This transducer is known as the piezoelectric micromachined ultrasound transducer (PMUT). The creation of PMUTs addresses some of the problems that traditional piezoelectric transducers faced and shares some of CMUTs’ advantages such as being able to integrate with IC and ease of fabricating a high-frequency array. PMUTs have reportedly been used for PAI purposes [108,109]. In comparison to CMUTs, PMUTs, like its counterpart, the conventional piezoelectric transducers still possess the inherent property of having a smaller bandwidth. In addition to that, there is a huge challenge in the designing and fabrication of PMUTs as their resonant frequency are very sensitive to the residual stress of the membrane [110]. Thus, fabricated PMUTs usually perform worse than what they can theoretically do. This potentially makes PMUTs a less attractive transducer.

## 4. Development of Capacitive Micromachined Ultrasound Transducer for Photoacoustic Imaging

The use of CMUTs for PAI has been researched for over a decade in mainly two types of application, the table top application with PACT and photoacoustic endoscopy. Various innovative ideas are being explored for PACT in areas such as 2D array [116,117,123,124], transparency [125,126,127], and multi-frequency [128,129,130]. In photoacoustic endoscopy, the designs being studied can be split into two different array configurations, phased [118,119] and ring arrays [120,121]. Using CMUTs as detectors for PAI has in fact moved from the lab to real-world biomedical application. A group of researchers in Japan has developed a CMUT based PAI mammogram [122].

### 4.1. Photoacoustic Computed Tomography with Capacitive Micromachined Ultrasound Transducer

#### 4.1.1. Two-dimensional Capacitive Micromachined Ultrasound Transducer

**Figure 3:**
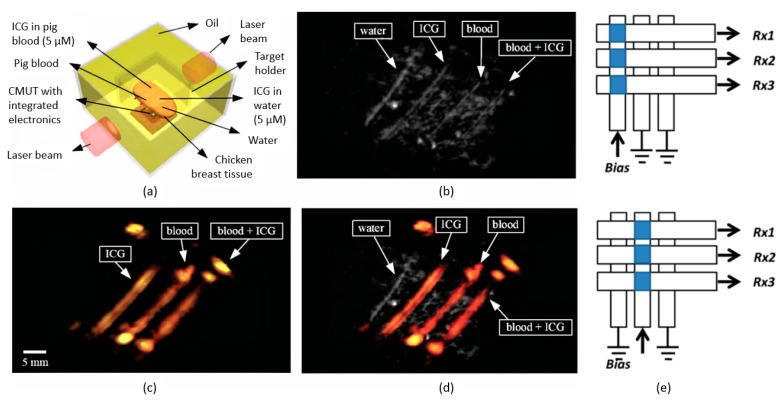
(**a**) Model of the chicken breast phantom, (**b**) ultrasonic imaging, (**c**) PAI, and (**d**) a combination of photoacoustic and ultrasonic imaging (© [2009] IEEE. Reprinted, with permission, from [117]); (**e**) working principle of top orthogonal to bottom electrode (TOBE) (© [2014] IEEE. Reprinted, with permission, from [124]).

An element of CMUT is made of numerous CMUT cells and multiple CMUT elements are used to form an array. A one-dimensional (1D) CMUT array is made of a row of CMUT elements. It uses electronic scanning for the rows and raster scanning for the columns or vice versa to form the image. With a 2D CMUT array, data can be acquired at a faster rate as both the rows and columns are imaged using electronic scanning. In 2005, Wygant et al. [116] was one of the first groups to have started investigating the use of CMUT array for PAI. A 5 MHz 2D 16 × 16 CMUT array was used to image polyethylene tubes filled with black ink and placed in a 2 × 2 × 3 cm block made of tissue-mimicking material. Both PAI and ultrasound imaging were used to image the phantom. PAI produced a better signal that requires lesser averaging to improve the SNR. This positive outcome opened the door to a new area of research for PAI. Vaithilingam et al. [117] went further by imaging a fishing line and chicken breast phantoms with the 2D 16 × 16 CMUT array. The fishing line phantom comprised of three 150 μm diameter transparent lines placed alternatingly between two 180 μm diameter black fishing lines. The chicken breast phantom was constructed with four polyethylene tubes that have an inner diameter of 1.19 mm positioned in chicken breast and covered with agar. The tubes were filled with four different solutions; deionized water, indocyanine green (ICG) solution in deionized water, pig blood, and ICG mixed with pig blood. Data were collected using ultrasound imaging and PAI on both phantoms. Ultrasound imaging was able to detect all the fishing lines and tubes in both phantoms, while with PAI, only the two black fishing lines and three tubes excluding the one containing deionized water in the chicken phantom were captured. The images taken from the ultrasound imaging and PAI is shown in Figure 3a–d The axial and lateral resolution acquired using PAI in the chicken phantom at 1.8 cm depth from the CMUT array were 300 μm and 648 μm, respectively.

Kothapalli et al. [123] pushed the limit of the penetration depth for PAI using CMUT array further to 5 cm. Horse hair embedded 2.2 cm, 3.1 cm, 4.1 cm, and 5.3 cm deep inside a chicken breast was imaged using a 5.5 MHz 2D 16 × 16 CMUT array. Because of scattering, the SNR decreased from 36 dB at 2.2 cm to 23 dB at 5.3 cm deep in the chicken breast. The calculated lateral resolution and axial resolution at 5.3 cm were approximately 720 μm and 370 μm, respectively. The group went on to investigate the sensitivity by varying the concentration of ICG positioned 5 cm deep in the tissue. The SNR showed a decreasing trend as the concentration decreased. The highest SNR of 37 dB was achieved from 10 μM of ICG while the lowest SNR of 13 dB was obtained from 100 nM of ICG.

Other than the design of CMUT, one important area to study is the addressing of large 2D CMUT array. A N × N CMUT array usually requires N × N number of connections to produce the image. This can create complications in the wiring of electrical circuits. To reduce the complexity of the electronics required for volumetric imaging, Chee et al. [124] introduced the top orthogonal to the bottom electrode (TOBE) architecture. TOBE involves biasing a column of the CMUT array and transmitting the signal along a row therefore, only N laser pulses and N channels are needed for imaging. Figure 3e illustrates the working principle of TOBE. A 3.7 MHz 2D 40 × 40 CMUT array was used to image three strands of hair placed 2 mm apart in oil. The calculated lateral and axial resolutions were 866 μm and 296 μm, respectively. This area of research to reduce the complexity of the electronic circuit is essential for CMUT array in real-time PAI.

#### 4.1.2. Transparent Capacitive Micromachined Ultrasound Transducer

There are two modes of transmission for PAI, forward and backward mode, which are also known as transmission and reflection mode. In clinical applications like endoscopy, backward mode transmission is a more practical approach. Moreover, it helps to eliminate blind spots. One of the challenges that PAI faces is the placement of the laser and the transducer to optimize the illumination of the sample. To address this issue, researchers are coming up with ways to create a transparent CMUT.

**Figure 4:**
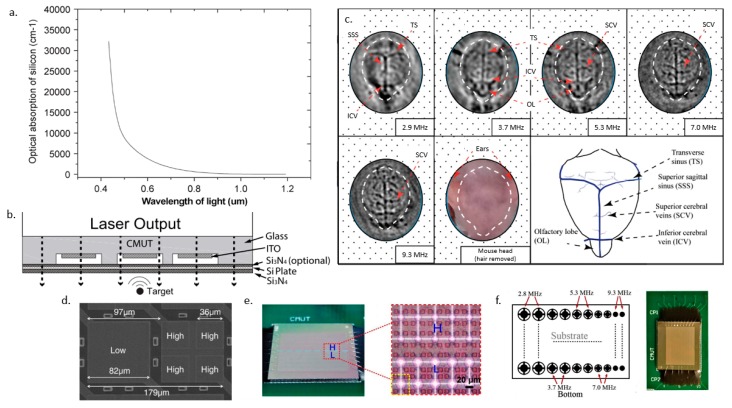
(**a**) Optical absorption of silicon under different wavelength (© [2010] IEEE. Reprinted, with permission, from [118]) and (**b**) structure of optically transparent CMUT (© [2018] IEEE. Reprinted, with permission, from [126]), (**c**) imaging of mouse brain using the different frequencies of the CMUT (© [2018] IEEE. Reprinted, with permission, from [130]), (**d**) interlaced CMUT (© [2017] IEEE. Reprinted, with permission, from [128]), (**e**) multi-band CMUT (Adapted with permission from ref [129], [The Optical Society]), (**f**) monolithic multiband CMUT with five frequencies (© [2018] IEEE. Reprinted, with permission, from [130]).

Chen et al. [125] designed a photoacoustic imager with a light source cascaded behind an infrared-transparent CMUT. The advantage of this design is that the realignment of the light source and the CMUT is not necessary. The device was used to image a metal wire phantom at varying distances from 2.3–4.5 cm. The measured light transmission of the 1.06 μm laser was about 12%. Zhang et al. [126] improved the transmission efficiency by fabricating a 1.4 MHz CMUT with a glass substrate and ITO bottom electrode, as shown in Figure 4b. The transmittance attained with this device was 30–70% at a wavelength of 700–900 nm. Two phantoms were used for PAI. The first phantom was a 0.7 mm diameter pencil lead immersed 1.5 cm deep in oil. In order to better mimic biological tissue, the second phantom used was made with a 2.3 mm inner diameter polyethylene tube filled with 50 μM ICG solution suspended in agar gel. In this work, the group mentioned that one of the reasons for low transmission in the visible light range is due to the use of silicon plate, which is not transparent in the visible light range as can be seen in Figure 4a. Li et al. [127] improved the transmission up to 82% in the visible light range by omitting the use of silicon in the fabrication process. The 2 MHz CMUT was characterized and found to have a noise equivalent sensitivity of 95 Pa when biased at 100 V.

#### 4.1.3. Multi-Frequency Capacitive Micromachined Ultrasound Transducer

Another research topic being explored for PAI is the multi-frequency CMUT array. A high-frequency transducer produces images with higher resolution and finer details while a low-frequency transducer experiences lesser attenuation and therefore produces images with higher SNR at deeper penetration depth [131]. With the huge frequency content produced by the tissue from the laser illumination, a multi-frequency CMUT array, where elements of different frequencies are arranged, can be used to capture both the outline and the fine details of the structure at varying depths.

Chee et al. [128] proposed an interlaced multi-frequency CMUT array for PAI as shown in Figure 4d. The lateral resolution obtained from imaging a strand of hair in oil immersion is 673 μm for the 1.74 MHz CMUT and 492 μm for the 5.04 MHz CMUT. The team went on to image a large target like methylene blue and a smaller target such as a strain of hair to analyze the effect of high and low frequency for spectroscopic PAI. The high-frequency element showed a higher SNR when imaging a small hair target, and the low-frequency element had a higher SNR imaging large methylene blue target.

In the same year, Zhang et al. [129] fabricated a multiband CMUT array with a radius of 15 μm for the low-frequency component at 3.7 MHz and 10 μm for the high-frequency component at 9.7 MHz. The multiband CMUT array is shown in Figure 4e. In vivo imaging of a zebrafish was done using this device, and a commercial ultrasound transducer. The outline structure of the zebrafish can be identified using the low-frequency transducer and the details of the zebrafish such as the swim bladder, the stripes, and mesenchymal tissues can be seen using the high-frequency CMUT element. This is reportedly the first in vivo PAI done using CMUTs.

Pun et al. [130] further developed the concept by incorporating five different frequencies of CMUTs 2.9 MHz, 3.7 MHz, 5.2 MHz, 7.0 MHz, and 9.3 MHz on a device, as shown in Figure 4f. The transducer was used to image the head of a mouse where with increasing frequency showed an increase in SNR which also results in a more detailed image of the brain of the mouse being captured as seen in Figure 4c. At 2.9 MHz, only the transverse sinus, superior sagittal sinus, and inferior cerebral vein were evident while at 9.3 MHz, on top of those blood vasculatures, superior cerebral vein and olfactory lobe can be seen.

The above experiments present different arrangements of the high and low-frequency elements for a multi-frequency CMUT array. Because of the complexity of the circuit and potential problems that might arise from the cross talk, imaging was done using raster scanning of only one CMUT element at a time. Indeed, a multi-frequency transducer is able to give us more information ranging from the minute details to the overall structure of the image. However, to fulfill this, future work would need to address possible problems associated with the complexity of the circuit and cross talk.

### 4.2. Photoacoustic Endoscopy with Capacitive Micromachined Ultrasound Transducer

One major limitation for PAI is the restriction in penetration depth because of optical scattering. As CMUT elements can be tightly packed to form the array, CMUT arrays are sufficiently small enough to be easily attached to catheters or endoscope. This brings the detector closer to the tissues, reducing scattering and increasing the resolution. Photoacoustic endoscopy is a change from the larger table top approaches and can be implemented with any of the three imaging modalities, PACT, OR-PAM, and AR-PAM in the two CMUT array configurations, phased and ring array.

#### 4.2.1. Phased Array Capacitive Micromachined Ultrasound Transducer

**Figure 5:**
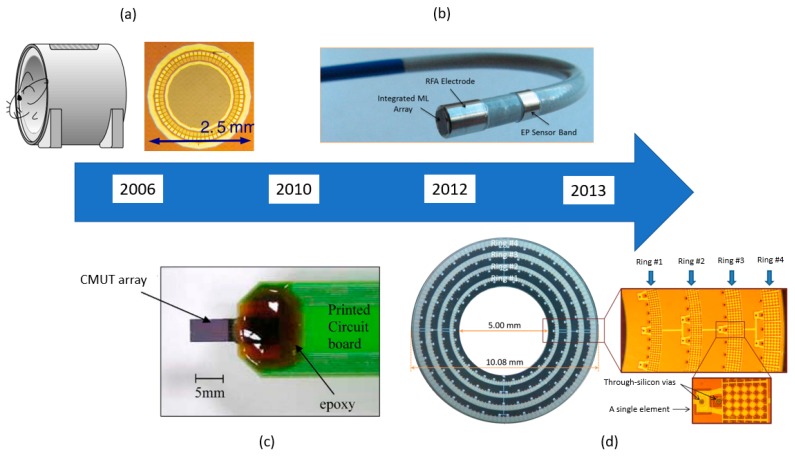
Timeline of CMUT designs for PAI endoscopes [118,119,120,121]; (**a**) inward-looking cylindrical transducer (© [2006] IEEE. Reprinted, with permission, from [120]); (**b**) 9F MicroLinear CMUT ICE catheter (© [2012] IEEE. Reprinted, with permission, from [119]); (**c**) miniature needle-shaped CMUT (© [2010] IEEE. Reprinted, with permission, from [118]); (**d**) integrated ring CMUT array (© [2013] IEEE. Reprinted, with permission, from [121]).

Phased and ring arrays are two popular configurations for photoacoustic endoscopy using CMUTs. Phased arrays are generally less complicated to design and fabricate as compared to ring arrays. Some research groups had come up with innovative designs for photoacoustic endoscopes using phased array. Chen et al. [118] designed a needle-shaped CMUT phased array which can be used for MIS applications, shown in Figure 5c. The needle-shaped design is made of 5 MHz CMUT elements with a diameter of 46 μm and is placed on a 2.8–5 mm by 8–18 mm silicon bar. PAI of a lobster nerve cord was done with one element on a rotating stage to collect data at multiple points. Since this design uses a CMUT that is transparent in the near-infrared region, there is minimal shadowing issue. This design showed the miniaturization capability of CMUTs, which is an important factor for MIS endoscopy applications.

Conventionally, CMUTs have been used mainly for ultrasound imaging. The previous work is done by Vaithilingam et al. [119] also showed the limitation of PAI, where it is unable to image transparent objects. Since both PAI and ultrasound imaging have different advantages, Nikoozadeh et al. [119] experimented using CMUT phased array for endoscopic imaging by integrating both PAI and ultrasound imaging into a catheter. The structure of the catheter is shown in Figure 5b. The 6.64 MHz CMUT array is able to image the outline of the tumor that is 15 mm deep in a phantom model of a mouse’s subcutaneous kidney.

#### 4.2.2. Ring Array Capacitive Micromachined Ultrasound Transducer

The circular view provided by a ring array gives a wider field of view and reduces the occurrence of reconstruction artifacts [120]. Moreover, a ring array design can make use of the empty spaces around the probe to optimize the space available. Vaithilingam et al. [120] designed one of the first CMUT ring array for PAI, an inward-looking cylindrical transducer array in 2006 as shown in Figure 5a. A 3 MHz CMUT ring array was used to image ink-filled polyethylene tube placed in a tissue-mimicking material. The axial and lateral resolution attained from the experiment was 350 μm and 4 mm. On top of the advantages of a circular view, as stated above, this inward-looking cylindrical transducer array also has the advantage of enclosing the target. This immensely helps to minimize the formation of motion artifacts and increase the image resolution.

Nikoozadeh et al. [121] proposed another interesting design where four CMUT ring arrays with different diameters and center frequencies are stacked together in a probe. A structure of this CMUT ring array is illustrated in Figure 5d. The frequencies of the rings are 16 MHz, 12 MHz, 8 MHz, and 6.5 MHz. In this paper, only 8 MHz and 6.5 MHz CMUT ring arrays were used to image a 13 mm spring and a custom wire phantom with ten 0.15 mm diameter nylon fishing lines. Both rings were able to produce images of the metal spring at 45 mm deep and the custom wire phantom at 15 mm deep. This design made use of the advantages of both multi-frequency and ring arrays to produce a photoacoustic endoscopy probe.

### 4.3. Industrialization of Capacitive Micromachined Ultrasound Transducer for Photoacoustic Imaging

Currently, Canon is rolling out its 3D real-time photoacoustic mammogram into the market. Canon has worked with Kyoto University in developing this system with experimental testing and clinical trials which can be found in research papers produced by the group. Asao et al. [122] had built a prototype of the photoacoustic mammogram with a CMUT-based receiver called PAM-02. A piezoelectric transducer was used in their initial prototype, PAM-01, but to increase the system’s imaging sensitivity, the team replaced it with a CMUT receiver. A more recent clinical trial was done with another of their prototype, PAI-04 [132]. PAI-04 is similar to its predecessor, PAI-03 with the addition of an ultrasound imaging system. PAI-04 was able to image the arterioles and venules of the palm, thigh, and breast with 500 4 MHz CMUT elements as the receiver. This is reportedly the first imaging done for tumor-related blood vessels in human cancer tissue that can achieve such fine details.

### 4.4. Challenges and Prospects of Using Capacitive Micromachined Ultrasound Transducer for Photoacoustic Imaging

Like many other micro-electro-mechanical systems (MEMS) devices, CMUTs also face the issue of dielectric charging where the built-up of charges causes the center frequency to be shifted [102]. This affects the performance of CMUTs and can potentially cause them to breakdown. Some proposed solutions to resolve this issue includes replacing the insulation layer with an insulator post [133], extending the insulation layer [134], and having a spacer below the membrane [135]. One of CMUTs’ main structure is the micron-scale thin membrane which can be easily damaged if it is not well protected. Encapsulation helps to minimize the damage, but there is a trade-off as a thicker encapsulation layer causes more acoustic attenuation which reduces CMUTs’ efficiency [102]. Another challenge that CMUT arrays face is crosstalk. Since the elements are placed on the same substrate, the vibration of one CMUT element inevitably affects the neighboring elements [102]. These unwanted vibrations result in the production of noise henceforth impairing the image quality. Researchers have been exploring various solutions to address the issue of crosstalk using device modeling [136] and also programmable waveforms [137].

With all the challenges and limitations of CMUT technology, the prospects of using CMUTs for PAI is promising. Currently, most of the CMUTs used for PAI applications operate in the lower frequency range, commonly less than 10 MHz. However, fabrication of CMUTs above 10 MHz has been demonstrated for use in ultrasound imaging [138,139,140]. For future development of CMUTs in PAI applications, one crucial aspect is to attain higher resolution imaging with higher frequency CMUTs. Another area to explore is the advancement of CMUTs for real-time 3D PAI applications. Even though 2D CMUT arrays have been fabricated, most results thus far have still relied on mechanically scanning. Operating a 2D CMUT array system without requiring this mechanical scanning may help to further push CMUTs in the direction of real-time 3D PAI applications. The ability to make a flexible CMUT array is another untapped domain for the future development of CMUTs for PAI. Besides being able to conform to different geometries, a flexible device can also provide a larger FOV for imaging applications. Various groups have shown the fabrication and potential application of flexible 2D CMUT arrays for ultrasound imaging [31,141,142]. A flexible 2D CMUT array would provide a strong candidate when compared with other techniques for MIS application where a small active area and large FOV is needed. It will also be immensely useful in large FOV PACT applications such as the imaging of brain [2,42,43] and breast cancer [45] or a virtual point transducer to enhance the FOV further and potentially reduce the SNR [4].

## 5. Conclusions

Overall, this review gives a brief outline of photoacoustic imaging (PAI), the basic principle of operation, and the different PAI modalities. PAI is an upcoming imaging technique that produces high-resolution images at deep penetration depth. In this paper, the more commonly used acoustic sensors for PAI, how they function, and their usage for different applications were discussed. CMUT stands out among the other acoustic sensors as it has the advantage of being fabricated into an array without much complication. This helps to shorten the imaging time and minimize the occurrence of motion artifacts. CMUTs can also be integrated with CMOS, which can help to maximize SNR so as to produce high-quality images. Finally, the development of CMUTs for PAI is presented along with the challenges and prospects of CMUTs for PAI. The potential in this area can be seen from the development of the photoacoustic mammogram system by Canon and Kyoto University. With the ability to be made for high resolution, deep penetration depth, larger FOV, and real-time imaging, there is a lot more room for CMUTs’ development for PAI applications.

## Figures and Tables

**Table 1:** The resolution, penetration depth, detector center frequency, and applications for the different photoacoustic imaging (PAI) modalities.

PAI Modalities	Typical Resolution Range	Typical Penetration Depth	Detector Center Frequency	Application
Photoacoustic computed tomography (PACT) [2,4,37,38,43,44]	>70 μm	70 mm	<10 MHz, but higher frequency detectors have been used	Suitable for applications such as functional imaging that requires imaging deeper with a larger FOV
Acoustic resolution photoacoustic microscopy (AR-PAM) [46,49,51,53,64]	>40 μm	11 mm or even up to several centimeters with contrast agents	Ranges from 2 MHz to 40 MHz, depending on whether a higher resolution or deeper penetration depth is desired	Generally used in reflection mode application
Optical resolution photoacoustic microscopy (OR-PAM) [15,46,48,57,58,59,60]	<10 μm	1–2 mm	>20 MHz	Useful for imaging smaller samples that are near the surface including the blood vessels, and cells

**Table 2:** The performance of different acoustic detectors along with their advantages and disadvantages in PAI applications.

Detectors	Resolution (μm)	Penetration Depth (mm)	Sensitivity	Advantages	Disadvantages
Fabry–Perot interferometer (FPI) [67,68,69,70,111,112]	<10	0.7–20	NEP: 80–300 Pa	- High-resolution image with small active area	- Challenging to fabricate into an array - Mechanical scanning - Smaller penetration depth
Micro-ring resonator (MRR) [81,82,113]	<10	0.002–2.2	NEP: 35–105 Pa	- Wide angular response - Low NEP over a wide frequency range	- Challenging to fabricate into an array - Mechanical scanning - Smaller penetration depth
Piezoelectric transducer (PZT) [98,114,115]	200	>30	SNR: 18–22 dB	- Most matured and readily available - Deeper penetration depth	- Opaque - Not CMOS compatible - Difficulty in fabricating high-density array
Capacitive micromachined ultrasound transducer (CMUT) [116,117,118,119,120,121,122,123,124,125,126,127,128,129,130]	>80	>50	SNR: 22–87 dB	- CMOS compatible - High-density arrays can be fabricated - Deeper penetration depth	- DC voltage is needed - Dielectric charging
